# Sexual and gender minority health in medical curricula in new England: a pilot study of medical student comfort, competence and perception of curricula

**DOI:** 10.1080/10872981.2018.1461513

**Published:** 2018-05-02

**Authors:** Nicole Sitkin Zelin, Charlotte Hastings, Brendin R. Beaulieu-Jones, Caroline Scott, Ana Rodriguez-Villa, Cassandra Duarte, Christopher Calahan, Alexander J. Adami

**Affiliations:** a Yale University School of Medicine, New Haven, CT, USA; b The Robert Larner, M.D. College of Medicine at the University of Vermont, Burlington, VT, USA; c Geisel School of Medicine at Dartmouth, Hanover, NH, USA; d School of Public Health and Tropical Medicine of Tulane University, New Orleans, LA, USA; e The Warren Alpert School of Medicine at Brown University, Providence, RI, USA; f Harvard Medical School, Boston, MA, USA; g University of Connecticut School of Medicine, Farmington, CT, USA

**Keywords:** Medical education, cultural literacy, sexual orientation, gender identity, sexual and gender minorities

## Abstract

**Background**: Sexual and gender minority (SGM) individuals experience high rates of harassment and discrimination when seeking healthcare, which contributes to substantial healthcare disparities. Improving physician training about gender identity, sexual orientation, and the healthcare needs of SGM patients has been identified as a critical strategy for mitigating these disparities. In 2014, the Association of American Medical Colleges (AAMC) published medical education competencies to guide undergraduate medical education on SGM topics.

**Objective**: Conduct pilot study to investigate medical student comfort and competence about SGM health competencies outlined by the AAMC and evaluate curricular coverage of SGM topics.

**Design**: Six-hundred and fifty-eight students at New England allopathic medical schools (response rate 21.2%) completed an anonymous, online survey evaluating self-reported comfort and competence regarding SGM health competencies, and coverage of SGM health in the medical curriculum.

**Results**: 92.7% of students felt somewhat or very comfortable treating sexual minorities; 68.4% felt comfortable treating gender minorities. Most respondents felt not competent or somewhat not competent with medical treatment of gender minority patients (76.7%) and patients with a difference of sex development (81%). At seven schools, more than 50% of students indicated that the curriculum neither adequately covers SGM-specific topics nor adequately prepares students to serve SGM patients.

**Conclusions**: The prevalence of self-reported comfort is greater than that of self-reported competence serving SGM patients in a convenience sample of New England allopathic medical students. The majority of participants reported insufficient curricular preparation to achieve the competencies necessary to care for SGM patients. This multi-institution pilot study provides preliminary evidence that further curriculum development may be needed to enable medical students to achieve core competencies in SGM health, as defined by AAMC. Further mixed methods research is necessary to substantiate and expand upon the findings of this pilot study. This pilot study also demonstrates the importance of creating specific evaluation tools to assess medical student achievement of competencies established by the AAMC.

## Introduction

Seventy percent of gender minority and over 50% of sexual minority individuals report experiencing harassment and discrimination while seeking healthcare [1]. Sexual and gender minority (SGM) individuals report substantial concerns about such past, and potential future, experiences. These concerns are linked to impaired patient-provider relationships and elevated rates of healthcare avoidance among SGM patients [1–4], which in turn contribute to health disparities between SGM and non-SGM patients [5,6]. Improving physician training about gender identity, sexual orientation, and the healthcare needs of SGM has been identified as a critical strategy for mitigating these disparities and improving the care delivered to SGM patients [6].

Physicians have historically received little to no required training on providing sensitive, competent care to SGM patients [6]. Seventy percent of allopathic medical school deans rate their institution’s curricular coverage of SGM-specific health topics as very poor, poor or fair [7]. Similarly, two thirds of allopathic and osteopathic medical students rate their institution’s SGM curricula as fair or worse [8]. In response to the need for SGM curricular development, in 2014 the Association of American Medical Colleges (AAMC) Advisory Committee on Sexual Orientation, Gender Identity and Sex Development (AXIS) published undergraduate medical education competencies pertaining to SGM populations. The stated goal of the competencies is to ‘serve as a primary resource for the medical education community to use in determining whether trainees can provide clinically sound, culturally competent care to these patient populations’ [6]. Research is burgeoning medical student learning outcomes related to SGM health, including attitudes, knowledge and competence. For example, in 2018, Beck et al. reported that student attitudes, knowledge, and preparedness to care for SGM patients were generally positive at four Midwestern medical schools (BECK 2018). Our investigation expands this and previous literature by reporting data from multiple medical schools and utilizing the AAMC competencies as the lens through which to explore student learning. Indeed, to the authors’ knowledge, this study is the first to date to explore self-reported comfort and competence ***explicitly*** within the framework of the AAMC SGM health competencies. These data promise to build upon and update earlier research [8] evaluating medical students’ comfort and preparedness in serving SGM patients. Medical student satisfaction with SGM content in the curriculum may function as a proxy of medical student evaluation of the overall efficacy of the curriculum as preparation for serving SGM patients.

This pilot study was designed to evaluate self-reported medical student comfort and competence in caring for SGM patients within the paradigm of the AAMC competencies, as well as satisfaction with SGM curricular content. The authors hypothesized that self-reported comfort and competence would be low across institutions, with modest improvements by ascending year, and that respondents would be unsatisfied with SGM curricular content.

The following terms pertaining to SGM individuals will be used in this manuscript:

- **Sexual minority**: individuals whose sexual orientation identity is anything other than heterosexual, or straight, and/or whose sexual behavior is not exclusively with individuals of the opposite binary gender (male or female).
- **Gender minority**: individuals whose internally felt gender identity and/or external gender expression is not congruent with the gender identity and/or expression associated with the sex assigned to them at birth.
- **Difference of Sex Development (DSD**): one of a variety of congenital conditions for which chromosomal, hormonal, and/or anatomic sex does not align with binary definitions of ‘male’ or ‘female’ (e.g. XXY syndrome, Primary adrenal insufficiency, Congenital adrenal hyperplasia)

## Methods

### Participants

The Northeast Medical Student Queer Alliance (NMSQA) is a collaborative organization representing medical students committed to SGM curricular reform from all ten allopathic medical schools in the New England census region :The Warren Alpert School of Medicine at Brown University; Boston University School of Medicine; Frank H. Netter MD School of Medicine at Quinnipiac University; Geisel School of Medicine at Dartmouth; Harvard Medical School; Tufts University School of Medicine; University of Connecticut School of Medicine; University of Massachusetts Medical School; University of Vermont College of Medicine; and Yale University School of Medicine. In December 2015, NMSQA initiated a pilot study of SGM learning outcomes in medical school curricula [9].

### Measures

The anonymous online survey (Appendix) was developed by medical student members of NMSQA to evaluate student comfort and competence related to the SGM health competencies outlined by the AAMC. As this study was a pilot project intended to gather preliminary data which could later be used for survey tool refinement, formal validity testing was not conducted.

Demographic data including sex assigned at birth, gender identity, sexual orientation, religiosity, and medical school attended were collected. The self-report questions were directly modeled on self-reported comfort questions routinely used on the AAMC’s Graduating Student Questionnaire [10]. Using the 4-point Likert items adapted from the AAMC Graduating Student Questionnaire [10], self-reported comfort and competence were evaluated for a selection of AAMC competencies [6]. These competencies were chosen by consensus as the fundamental competencies addressed by any and all medical school curricula designed to promote high-quality care to SGM patients, regardless of the complexity of the curricula. Four-point Likert items (1 = not at all; 4 = completely) designed with the assistance of the Yale School of Medicine Teaching and Learning Center were used to evaluate student perceptions of the adequacy of curricular preparation for serving SGM patients and adequacy of curricular coverage of SGM topics.

### Survey implementation

To access the survey, all respondents were required to review and electronically grant informed consent for participation. The Yale Human Subjects Committee deemed this study exempt from review (HSC #1,505,015,780) as this study involved minimal risk to participants, no personal health information was collected, and all participants were anonymous. The Dartmouth College Institutional Review Board approved dissemination of the survey to its medical students. Regulatory approval for survey dissemination was not required at other institutions. All respondents were eligible to participate in a raffle for ten $25 Amazon gift cards

The survey was distributed to medical students via email, social media platforms requiring school enrollment, and school-sponsored newsletters. The inclusion criteria included active enrollment at a NMSQA member school, as of May 2015. The web-based survey was available from 3 May 2015 to 13 August 2015. 910 students completed some or all of the survey between May and September 2015. The study cohort represents a convenience sample, with an overall response rate of 21.2%. Due to institution-specific factors, the survey was not administered at one institution, and thus, no responses were received from one institution. Incomplete responses (<70% items completed) were excluded. Responses submitted within two minutes of survey initiation were excluded from analysis; this threshold was approximated as a minimum threshold for reliable completion of the survey NMSQA members. All individual responses were anonymous and aggregate school data were de-identified.

### Statistical analysis

The primary outcomes were: (1) self-reported comfort in providing care to SGM patients; (2) self-reported competence in providing care to SGM patients; and (3) curriculum assessment. The index for medical student comfort treating SGM patients was calculated as the mean of six 4-point Likert items (1 = not comfortable, 4 = very comfortable) querying comfort with AAMC competencies. The index for self-reported competence in treating SGM patients was calculated as the mean of 15 4-point Likert items (1 = not competent, 4 = very competent) querying self-perceived competence with AAMC competencies. Perceived adequacy of the curriculum was calculated as the mean of six 4-point Likert items (1 = strongly disagree, 4 = strongly agree) describing curricular effectiveness at preparing students to care for SGM patients. For all composite measures, the mean index was calculated for completed items; incomplete items were not included. Cronbach’s alpha statistic was calculated for each index measure to determine internal reliability of the composite items. As all alpha statistics were above 0.70 (comfort: 0.8673, competence: 0.9397, satisfaction with curriculum: 0.9235), each composite measure was internally reliable.

Summary statistics were computed among all participants and stratified independently by class year, gender identity, and sexual orientation. The Kruskal–Wallis test by ranks (a non-parametric one-way analysis of variance) was performed to identify differences by class year, gender identity and sexual orientation. A post-hoc Tukey analysis was performed (Tukey HSD test) as appropriate to conduct pairwise comparisons and determine how outcomes varied. All statistical tests were evaluated at a *p = *0.05 significance level. All analyses were performed using Stata (StataCorp, College Station, TX).

## Results

Six hundred and fifty-eight medical students from nine institutions were included in the final analytic sample (Table 1). First year medical students (34.1%) were most often represented, with the remaining respondents split nearly equally among years two through four. Nearly 60% of participants reported sex assigned at birth as female. Fifty-nine percent of participants reported a gender identity of ‘woman,’ 38.3% ‘man’, and 0.6% ‘genderqueer.’ Approximately 80% of participants identified as heterosexual, 7.0% as bisexual, 6.4% as gay, 2.6% as lesbian, 2.1% as queer, and 0.9% as other. A majority of students were either ‘not at all religious’ (44.2%) or ‘slightly religious’ (19.9%).

**Table 1.** Demographic characteristics of participating medical students (*N* = 658).

Class Year	*N* (%)
First (M1)	224 (34.1)
Second (M2)	147 (22.4)
Third (M3)	143 (21.6)
Fourth (M4)	144 (21.9)
**Sex assigned at birth**	
Male	252 (38.3)
Female	392 (59.6)
Missing response	14 (2.1)
**Gender identity**	
Male	252 (38.3)
Female	388 (59.0)
Genderqueer	4 (0.6)
Missing response	14 (2.1)
**Sexual orientation**	
Heterosexual	516 (78.4)
Bisexual	46 (7.0)
Gay	42 (6.4)
Lesbian	17 (2.6)
Queer	14 (2.1)
Other	6 (0.9)
Missing response	17 (2.6)
**Religiosity**	
Not at all	291 (44.2)
Slightly	131 (19.9)
Moderately	90 (13.7)
Quite	84 (12.8)
A whole lot	47 (7.1)
Missing response	15 (2.3)

The median composite comfort index for all students was 3.0 ± 0.7 (range 1–4), which corresponds to feeling ‘somewhat comfortable’ caring for SGM patients (Table 2). This value did not differ by class year (*p *= 0.51). More students were comfortable treating sexual minorities (92.7%) than gender minorities (68.4%; Table 3).

**Table 2.** Composite values for learning outcomes, overall, and by class year.

Outcome	Composite Index Median (SD)					K-wallis	
	Overall (*n* = 658)	M1 (*n* = 224)	M2 (*n* = 147)	M3 (*n* = 143)	M4 (*n* = 144)	Chi-square	*p*-value
Comfort	3.0 (0.7)	3.0 (0.7)	3.0 (0.7)	3.0 (0.6)	3.0 (0.6)	2.31	0.51
Competence	2.4 (0.7)	2.3 (0.7)	2.3 (0.7)	2.5 (0.6)	2.5 (0.7)	14.71	0.002
Curriculum	2.3 (0.8)	2.2 (0.8)	2.3 (0.8)	2.7 (0.8)	2.5 (0.8)	16.14	0.001

**Table 3.** Medical student self-reported comfort and competence, individual survey items on AAMC competencies.

Comfort Items	Number of Respondents *N* (%)			
	Not comfortable	Somewhat not comfortable	Somewhat comfortable	Very comfortable
Treat sexual minority (e.g., queer, bisexual, lesbian, gay) patients	9 (1.4)	39 (5.9)	234 (35.6)	375 (57.1)
Treat gender minority (e.g., transmasculine, transfeminine, genderqueer) patients	40 (6.1)	168 (25.6)	281 (42.8)	168 (25.6)
Discussing sexual orientation (that is, an individual’s sexual attraction, sexual partners, and sexual orientation identity, such as LGBQ) with patients	17 (2.6)	71 (10.8)	231 (35.1)	339 (51.5)
Discussing sexual practices with sexual and gender minority patients (e.g., bottom/top, sex toy use, dental dam use)	67 (10.2)	184 (28.0)	237 (36.0)	170 (25.8)
Discussing gender identity (that is, individuals’ internal perception or sense of their own gender) with patients	34 (5.2)	141 (21.5)	236 (36.0)	244 (37.3)
Discussing sexual and gender minority-specific health topics (e.g., hormone therapy, reciprocal *in vitro* fertilization, safe sex practices for sexual minorities)	78 (11.9)	191 (29.2)	214 (32.7)	172 (26.3)
	Number of Respondents *N* (%)			
Competence Items	Not competent	Somewhat not competent	Somewhat competent	Very competent
Sensitively interview patients about sexual orientation, sexual history, and sexual practices	17 (2.6)	86 (13.1)	295 (44.7)	262 (39.7)
Sensitively interview transgender and GNC patients about their gender identities, health and risk behaviors, and physical anatomy	71 (10.8)	221 (33.7)	249 (37.8)	118 (17.9)
Describe treatment options for transgender patients, including pre-pubertal hormone block, hormone therapy and surgeries	281(42.7)	225 (34.0)	102 (15.4)	53 (8.0)
Describe treatment options for patients born with DSD, differentiating between elective and non-elective therapies and surgeries for the most common DSD conditions	316 (47.8)	219 (33.2)	85 (12.9)	40 (6.1)
Describe key screening recommendations for sexual and gender minorities	184 (27.8)	221 (33.4)	178 (26.9)	78 (11.8)
Define and describe the differences between the following: sex and gender; gender expression and gender identity; and between gender discordance, gender nonconformity and gender dysphoria	65 (9.9)	170 (25.9)	229 (34.9)	193 (29.4)
Describe etiologies of atypical sex development	168 (25.5)	235 (35.7)	179 (27.1)	77 (11.7)
Describe historical, political, sociocultural, and institutional factors that contribute to the development and maintenance of health disparities among LGBTQ patients, GNC patients and patients born with DSD, including historical and current provider practices (e.g., reparative therapy)	130 (19.7)	249 (37.8)	182 (27.6)	98 (14.9)
Identify and address communication patterns in the health care setting that adversely affect care of LGBTQ, GNC, and DSD patients	96 (14.6)	219 (33.2)	231(35.1)	113 (17.2)
Describe how patients’ and families’ healing traditions and beliefs might shape reactions to diverse forms of sexuality, sexual behavior/orientation, gender identity, gender expression, and sex development	88 (13.4)	195 (29.6)	257 (39.0)	119 (18.1)
Employ appropriate consent and assent practices for disclosure of gender, sexuality, and sex issues in a clinical setting	100 (15.2)	195 (29.7)	222 (33.8)	140 (21.3)
Describe the special challenges faced by health professionals who identify with one or more of the following populations: LGBTQ, GNC, DSD	104 (15.9)	223 (34.0)	219 (33.4)	110 (16.8)
Describes the strategies that can be used to enact reform within existing health care institutions to improve care to LGBTQ, GNC, and DSD patients	150 (22.9)	267 (40.7)	173 (26.4)	66 (10.1)
Describe the special legal and policy issues that affect LGBTQ, GNC, and DSD patients	163 (24.8)	253 (38.5)	170 (25.8)	72 (10.9)
Identify your own implicit biases which impact the care delivered to LGBTQ, GNC, and DSD patients and develop strategies to mitigate their impact	31 (4.7)	139 (21.2)	317 (48.2)	171 (26.0)

The median composite competence index for all students was 2.4 ± 0.7 (range 1–4), corresponding to a response between ‘somewhat not competent’ and ‘somewhat competent’ (Table 2). Students felt most competent in their ability to sensitively interview patients about sexual orientation, sexual history, and sexual practices (84.4% somewhat or very competent, Table 3). Respondents reported the least competence in describing treatment options for transgender patients (23.4% somewhat or very competent) and for patients born with a difference of sex development (19.0% somewhat or very competent).

With regard to students’ perceptions of their respective medical school SGM-related curriculum, the mean composite index was 2.3 ± 0.8 (Table 2), which reflects a moderately negative perception of curricula. Notably, the majority of students did not believe that their curriculum adequately prepared them to comfortably and competently care for SGM patients (55.9%) or adequately covered SGM-specific health topics (60.3%, Table 4).

**Table 4.** Medical student self-reported satisfaction with SGM curricular content.

Survey Items Capturing Medical Student Evaluation of Curriculum	Number of Respondents *N* (%)			
	Strongly disagree	Somewhat disagree	Somewhat agree	Strongly agree
The formal curriculum at my school has adequately prepared me to comfortably and competently serve sexual and gender minorities	123 (18.8)	244 (37.2)	217 (33.1)	72 (11.0)
The formal curriculum at my school adequately covers sexual orientation diversity	112 (17.1)	195 (30.0)	215 (32.7)	135 (20.6)
The formal curriculum at my school adequately covers gender diversity	129 (19.7)	199 (30.3)	215 (32.8)	113 (17.2)
The formal curriculum at my school adequately covers health disparities among sexual and gender minorities	126 (19.2)	225 (34.4)	205 (31.3)	99 (15.1)
The formal curriculum at my school adequately covers sexual and gender minority-specific health topics	137 (20.9)	259 (39.4)	184 (28.0)	77 (11.7)
Over the course of my medical education, I have had the opportunity to practice interacting with sexual and gender minority patients	131 (20.0)	204 (31.2)	206 (31.5)	113 (17.3)

Students’ self-reported competence (*p *= 0.002) and assessment of curricular incorporation of SGM healthcare topics (*p *= 0.001) varied by class year (Table 2). Tukey post-hoc testing demonstrated notable differences between first- and third-year students: third-year students reported greater competence working with SGM patients (*p *= 0.002) and thought their curricula had better coverage of SGM topics (*p *= 0.002). Similar differences were found between first- and fourth-year students, with fourth-year students reporting greater competence working with SGM patients (*p *= 0.01). No other significant differences by class were noted.

Furthermore, the primary outcomes (comfort, competence, and perceived adequacy of SGM curricula) differed by gender identity (Table 5). Notably, male participants, compared to their female counterparts, reported increased comfort (median 3.2 versus 3.0, *p *= 0.04), and satisfaction with SGM-curricular content (2.7 versus 2.2, *p *= 0.001). Differences were also observed by sexual orientation (Table 6). Compared to non-heterosexual identified respondents, heterosexual participants reported increased satisfaction with SGM curricular content (median 2.7 versus 2.2, *p *= 0.006), whereas sexual minority respondents reported increased comfort (3.3 versus 3.0, *p *< 0.001) and competence (2.7 versus 2.3, *p *< 0.001).

**Table 5.** Differences in composite outcomes by self-reported gender identification.

Outcome	Composite Index Median (SD)			K-wallis	
	Overall (*n* = 658)	Male (*n* = 252)	Female (*n* = 388)	Chi-sqaure	P-value
Comfort	3.0 (0.7)	3.2 (0.6)	3.0 (0.7)	4.06	0.04
Competence	2.4 (0.7)	2.5 (0.8)	2.4 (0.6)	7.17	0.007
Curriculum	2.3 (0.8)	2.7 (0.8)	2.2 (0.8)	32.46	0.001

Aggregate responses for genderqueer participants (*N* = 4) are not presented given group size.

**Table 6.** Differences in composite outcomes by self-reported sexual orientation.

Outcome	Composite Index Median (SD)			K-wallis	
	Overall (*n* = 658)	Heterosexual (*n* = 516)	LGBQ (*n* = 125)	Chi-sqaure	*p*-value
Comfort	3.0 (0.7)	3.0 (0.7)	3.3 (0.6)	15.44	<0.001
Competence	2.4 (0.7)	2.3 (0.7)	2.7 (0.6)	26.11	<0.001
Curriculum	2.3 (0.8)	2.7 (0.8)	2.2 (0.8)	7.12	0.006

The group ‘LGBQ’ includes: lesbian (17), gay (42), bisexual (46), queer (14), and other (6).

## Discussion

Medical student respondents to this pilot survey of nine of the ten New England allopathic medical schools (response rate: 21%) endorsed moderate self-reported comfort but limited self-reported competence in caring for SGM patients. The discrepancy between self-reported comfort and self-reported competence may reflect a distinction between provider affect (i.e. comfort) related to caring for SGM patients, and provider knowledge and skills necessary to competently care for SGM patients. Differences in competence and perception of curricular effectiveness were observed based on gender identity, sexual orientation, and class year. Over half of the participants reported inadequate preparation to serve SGM patients and inadequate coverage of SGM topics within current medical curricula.

Across institutions, the majority of respondents reported moderate comfort treating SGM patients, mirroring prior research documenting positive attitudes among osteopathic medical students toward treating sexual minorities [11] and recent reductions in overt anti-SGM sentiment in the general population, particularly among individuals with advanced education [12,13]. Self-reported comfort and competence were greater among sexual minority medical students compared to their heterosexual peers, which may be partially attributable to the high rates of involvement in SGM professional work reported by SGM medical trainees and providers [14] and to the impact of personal experience with SGM health issues. Additionally, male-identified respondents reported greater competence and satisfaction with curricular preparation than female-identified respondents. This trend parallels previously reported gender disparities in self-esteem and self-assessment: male medical students overestimate competence and endorse greater self-esteem, while female medical students underestimate performance and report less self-esteem [15,16]. This discrepancy may also reflect the social privilege of male identity in the heteronormative, pro-masculine culture documented in medical education [17,18]. Socially privileged groups exhibit greater implicit bias than marginalized groups [19], potentially inhibiting appropriate recognition of SGM health needs and motivation to develop SGM health knowledge and skills.

The vast majority of participants (92.7%) indicated comfort treating sexual minority patients. However, a minority (31.7%) of respondents expressed comfort caring for gender minority patients. Similarly, more than 25% of students were somewhat not or not comfortable discussing gender identity and more than 40% of students were somewhat not or not comfortable discussing gender minority health topics such as hormone therapy. Student discomfort with gender-related topics paralleled low levels of self-reported competency regarding sex and gender-related issues. Students reported the least competence with interviewing patients about gender identity, detailing treatment options for gender minorities and describing the etiologies of differences of sex development. These findings are consistent with published data showing that gender minorities report limited provider cultural competency, frequent denial of needed healthcare services, and overt harassment in healthcare settings [1,3,4,20]. Similarly, individuals born with differences of sex development have distinct medical needs that are often inadequately addressed or addressed in a way that enforces a binary model of sex and gender, provoking lifelong psychological trauma [21–23], and/or physical sequelae [6,24]. Our findings suggest that enhanced provider training to competently and sensitively serve these particular populations is needed.

More than half of the respondents in this study reported that their medical school curriculum did not adequately prepare them to serve SGM patients. While the composite index of perceived curricular effectiveness increased with class year, the majority of third- and fourth-year students still reported that their formal curriculum provided inadequate preparation. This trend may suggest that dissatisfaction during the pre-clinical years stems from an absence of salient SGM topics in the curriculum, which is only slightly ameliorated by clinical experiences and other learning opportunities over time.

Despite the perceived ineffectiveness of formal curricula, third- and fourth-year medical students reported greater competence compared to first-year students. This increase suggests that some aspect of undergraduate medical education is contributing to increased SGM healthcare knowledge and increased self-reported competence over the course of medical school. Of note, class year-related increases in competence were most evident in skills required for general patient care, such as patient interviewing and informed consent. Increases in competence with increasing class year may thus reflect a general advancement of clinical skills, as opposed to a true improvement with SGM health competencies.

### Limitations

This pilot study provides preliminary data about medical student learning outcomes related to the AAMC SGM competencies. However, it is limited by reliance on self-reported comfort and competency, which are imperfect measures of learning outcomes. For example, current research has suggested that up to 98% of providers endorse willingness to care for SGM patients [25], but a majority of SGM patients [1] report discrimination in healthcare, suggesting that providers may overestimate their competence in serving SGM patients. Similarly, nearly three-fourths of respondents in our study reported some degree of competence in identifying and developing strategies to mitigating their personal biases; however, a 2015 study of 4,000 medical students documented explicit anti-sexual minority bias in more than 40%, and implicit anti-sexual minority bias in more than 80%, of respondents [26]. The disparity between self-reported competence and related objective metrics in a comparable cohort suggests that respondents may have over-estimated their competence, or alternatively, may have mistaken comfort for competence. Given the pilot nature of this study, no formal validity testing was conducted, limiting interpretation of these data, particularly as self-reported measures may be affected by social desirability bias. Validity testing will be important for subsequent, more expansive follow-up studies on medical student learning about SGM health.

This study may further overestimate composite learning outcomes due to the disproportional representation of SGM medical students among the respondent pool, and the enhanced self-reported competence of sexual minority respondents comparted to heterosexual respondents. Approximately 20% of respondents self-identified as SGM, compared to 2 to 4% of individuals in the general population and 6% of first-year medical students [26–27].

Despite these limitations, this pilot study suggests the existence of a significant educational gap and a need for enhanced competency related to the care of SGM patients. A national assessment of medical student comfort and competence in treating SGM patients, ideally including qualitative and quantitative metrics, is needed to build upon and extend these findings. Such a study would be enhanced by a larger sample size and by including objective measures of learning outcomes.

Until such a study can be conducted, the present report of self-assessed comfort, competence and perception of curricular coverage may serve as a preliminary indicator of key areas for improvement in medical curricula. Recognizing the limitations of the present study, the authors recommend that medical curricula should prioritize topics for which notable shortcomings have been identified in current practice, as well as embed means to assess student competency in curricular activities. The following recommendations are issued with these caveats:

1. SGM health broadly remains an urgent topical area for curricular development and enhancement. In particular, curricular development to prepare students for serving gender minority patients and patients born with a difference of sexual development appears to be needed desperately. Holistic integration of SGM content in the curriculum, exposure to SGM patients and exposure to SGM topics in clinic encounters increase knowledge of SGM health and positively impact attitudes toward SGM patients [24,26,28,29]. Educators may expedite and facilitate curricular development by accessing the numerous resources developed by the AAMC (including clinical vignettes and webinars) [30] and the expanding library of peer-reviewed instructional materials on the AAMC’s MedEd Portal [15]. Additionally, educators may benefit from collaboration with institutions that have already begun the process of SGM content development and integration in the medical curriculum [31–33].
2. A substantial portion of respondents reported difficulties performing specific competencies related to SGM patient care. A more granular approach to developing, evaluating, and delivering formative feedback on specific competencies may therefore be a more effective approach for SGM curriculum delivery and evaluation. As self-reported comfort does not significantly vary with perceived competence, it likely should not be used as the primary endpoint for curriculum evaluation.
3. Student development of SGM-specific competencies seems to be linked to development of general medical competencies. Critical evaluation of student learning outcomes must be contextualized within the broader framework of learner development so as to detect when general learner development may mask persistent deficiencies in SGM health knowledge and competency.

## Conclusions

This pilot study indicates that a sample of medical students at allopathic medical schools in New England report limited competence with SGM healthcare competencies and perceive formal curriculum as inadequately preparing them to care for SGM patients. These data provide a snapshot of learning outcomes in the New England region. As provider attitudes and behaviors toward SGM patients and medical students’ experiences related to SGM health issues may vary significantly by region, additional research is needed to evaluate curricular needs in different geographic regions [24,28,34–40]. Assessment of objective, rather than self-reported, learning outcomes will also be a critical component of future research in this area.

In combination with prior research describing limited curricular coverage and integration of SGM health content [7,8], our findings suggest that further curricular development and medical education research is needed to appropriately prepare medical students to serve all patients, regardless of sexual orientation or gender identity, and to help mitigate the health disparities suffered by SGM patients.

## Geolocation information

This study was disseminated to students located in Vermont, New Hampshire, Massachusetts, Rhode Island and Connecticut.

## Appendix

Sexual Orientation,Gender Identity and Sex Development in Medical Curricula

Consent for Participation in a Research Project IRB Protocol # 1,505,015,780 Evaluating Sexual and Gender Minority Health in Medical School CurriculaNicole Sitkin, MD Candidate (nicole.sitkin@yale.edu) John Encandela, PhD (john.encandela@yale.edu) Purpose: You are invited to participate in a research study designed to investigate the degree to which medical school curricula enhance medical student knowledge, competence and comfort serving the health needs of sexual and gender minorities (SGM), including individuals who identify as lesbian, gay, bisexual, transgender or queer (LGBTQ), gender non-conforming (GNC) individuals, and individuals born with disorders or differences of sex development (DSD). All medical students at the following institutions are eligible to participate in this survey study: Alpert Medical School at Brown University Boston University School of Medicine Frank H. Netter MD School of Medicine at Quinnipiac University Geisel School of Medicine at Dartmouth Harvard Medical School Tufts University School of Medicine University of Connecticut School of Medicine University of Massachusetts Medical School University of Vermont College of Medicine Yale University School of Medicine We encourage eligible medical students of all years, sexes, sexual orientations, and gender identities to participate in this survey. Procedures: Participation in this study will involve completing an online survey. We anticipate that your involvement will require approximately 10 min. You will be eligible to enter a raffle for one of ten $25 Amazon gift cards following completion of the survey. Risks and Benefits: Participation in this study involves very minimal risk. There is a slight chance that you may feel uncomfortable answering some of the questions in the survey. Only two questions on this survey require an answer. One of these questions confirms your consent to participate in this research study, and the other question confirms your eligibility to participate in this study. You can decide to skip any other questions that you do not want to answer. You may withdraw from the study at any time. If you want to stop completing the survey, simply close the survey. While you may not personally benefit from participating in this study, the information you provide will yield important insights into the general state of medical curricula on SGM health among Northeast medical schools, and to lay the foundation for an improved understanding of key areas for curricular development and reform. Confidentiality: All of your responses will be completely anonymous. No identifying data of any kind will be collected. At the completion of the survey, you will have the opportunity to provide your name and e-mail to be entered into a raffle. You contact details cannot and will not be linked in any way to your responses on this survey. Voluntary Participation: Your participation in this study is voluntary. You are free to decline to participate, to end your participation at any time for any reason, or to refuse to answer any individual question. Refusing to participate will involve no penalty. Questions: If you have any questions about this study, you may contact the investigators, Nicole Sitkin (nicole.sitkin@yale.edu; 203–785-5466) and John Encandela (john.encandela@yale.edu; 203–785-5466). If you would like to talk with someone other than the researchers to discuss problems or concerns, to discuss situations in the event that a member of the research team is not available, or to discuss your rights as a research participant, you may contact the Yale University Human Subjects Committee, Box 208,010, New Haven, CT 06520–8010, 203–785-4688, human.subjects@yale.edu. Additional information is available at http://www.yale.edu/hrpp/participants/index.html Agreement to Participate: By clicking the box labeled ‘I Consent’ below, I certify that I have read the above information, that all of my questions about the study have been answered to my satisfaction and that I agree to participate in this study.

I consent (1)

Which medical school do you attend? You must be a medical student at one of the following medical schools in order to be eligible to participate in this survey.

Alpert Medical School (1)

Boston University School of Medicine (2)

Frank H. Netter MD School of Medicine at Quinnipiac University (9)

Geisel School of Medicine at Dartmouth (3)

Harvard Medical School (4)

Tufts University School of Medicine (5)

University of Connecticut School of Medicine (6)

University of Massachusetts Medical School (10)

University of Vermont College of Medicine (7)

Yale University School of Medicine (8)

For the following tasks, please indicate how comfortable you feel in achieving the stated task on a scale of 1–4. (1 = not comfortable; 2 = somewhat not comfortable; 3 = somewhat comfortable; 4 = very comfortable)

**Table:**

	1 (1)	2 (2)	3 (3)	4 (4)
Treating sexual minority (e.?g. queer, bisexual, lesbian, gay) patients (1)				
Treating gender minority (e.?g. transmasculine, transfeminine, genderqueer) patients (2)				
Discussing sexual orientation (i.?e., an individual’s sexual attraction, sexual partners, and sexual orientation identity, such as LGBQ) with patients (3)				
Discussing sexual practices with sexual and gender minority patients (e.?g. bottom/top, sex toy use, dental dam use) (4)				
Discussing gender identity (i.?e., individuals’ internal perception or sense of their own gender) with patients (5)				
Discussing sexual and gender minority-specific health topics (e.?g. hormone therapy, reciprocal *in vitro* fertilization, safe sex practices for sexual minority women etc.) (6)				

For the following tasks, please indicate how competent you feel in achieving the stated task on a scale of 1–4 (1 = not competent; 2 = somewhat not competent; 3 = somewhat competent; 4 = very competent).

**Table:**

	1 (1)	2 (9)	3 (10)	4 (11)
Sensitively interview patients about sexual orientation identity, sexual history and sexual practices. (1)				
Sensitively interview transgender and GNC patients about their gender identities, health and risk behaviors, and physical anatomy. (2)				
Describe the treatment options for transgender patients, including pre-pubertal hormone block, hormone therapy and surgeries. (3)				
Describe the treatment options for patients born with DSD, differentiating between elective and non-elective therapies and surgeries for the most common DSD conditions. (4)				
Describe key screening recommendations for sexual and gender minorities. (5)				
Define and describe the differences between the following: sex and gender; gender expression and gender identity; and between gender discordance, gender nonconformity, and gender dysphoria. (6)				
Describe the main etiologies of atypical sex development. (7)				
Describe the historical, political, sociocultural and institutional factors that contribute to the development and maintenance of health disparities among LGBTQ patients, GNC patients, and patients born with DSD, including historical and current provider practices (e.?g. reparative therapy). (8)				
Identify and address communication patterns in the health care setting that adversely affect care of LGBTQ patients, GNC patients, and patients born with DSD. (9)				
Describe how patients’ and families’ healing traditions and beliefs might shape reactions to diverse forms of sexuality, sexual behavior, sexual orientation, gender identity, gender expression, and sex development. (10)				
Employ appropriate consent and assent practices for disclosure of gender, sexuality and sex issues in a clinical setting. (11)				
Describe the special challenges faced by health professionals who identify with one or more of the following populations: LGBTQ, GNC, DSD. (12)				
Describe strategies that can be used to enact reform within existing health care institutions to improve care to LGBTQ patients, GNC patients, and patients born with DSD. (13)				
Describe the special legal and policy issues (e.?g. insurance limitations, lack of partner benefits, visitation and nondiscrimination policies) that affect LGBTQ patients, GNC patients, and patients born with DSD. (14)				
Identify your own implicit biases which impact the care delivered to LGBTQ patients, GNC patients, and patients born with DSD, and develop strategies to mitigate the impact of these biases. (15)				

To the best of your knowledge, please indicate whether each statement is ‘True’ or ‘False.’ You may also select ‘I do not know’ if you are unable to determine if the statement is ‘True’ or ‘False.’

**Table:**

	True (1)	False (2)	I do not know (3)
LGBTQ people mostly only experience sexual health-related disparities (eg. HIV/AIDS) (1)			
Transgender men may need pap smears. (2)			
LGBTQ individuals are more likely to report mental health problems (such as anxiety and depression). (3)			
Smoking is more prevalent among sexual minority women, putting them at greater risk for certain respiratory diseases. (4)			
All men who have sex with men are gay. (5)			
Suicidal ideation and attempted suicide are just as common among heterosexual, cisgender individuals as among LGBT individuals. (6)			
LGBTQ people experience a wide variety of disparities in risk and disease compared to their non-LGBTQ peers. (7)			
Some individuals exhibit genetic, hormonal or physiological phenotypes that do not fit into a strict sex-binary (i.?e. male and female). (8)			
Lesbians do not need routine pap smears, since they do not have sexual relations with men. (9)			

Please rate on a scale of 1 to 4 how strongly you agree or disagree with each statement (1 = strongly disagree; 2 = somewhat disagree; 3 = somewhat agree; 4 = strongly agree).For this question, ”formal curriculum” refers to medical school learning activities which are integrated in the standard M1, M2, M3 or M4 curriculum, and that are organized and run by medical school administrators and/or faculty. Learning activities organized or run by medical students or medical student groups do not qualify as formal curriculum for this question.

**Table:**

	1 (1)	2 (2)	3 (3)	4 (4)
The formal curriculum at my school has adequately prepared me to comfortably and competently serve sexual and gender minority patients (1)				
The formal curriculum at my school adequately covers sexual orientation diversity. (2)				
The formal curriculum at my school adequately covers gender diversity. (3)				
The formal curriculum at my school adequately covers health disparities among sexual and gender minorities. (4)				
The formal curriculum at my school adequately covers sexual and gender minority-specific health topics. (5)				
Over the course of my medical education, I have had the opportunity to practice interacting with sexual and gender minority patients (6)				

Thank you for participating in this survey! You have now completed all of the curriculum questions. Only a few brief demographics questions to go!

Respondent Demographics (10 questions)

What is your year in medical school?(In other words, please indicate the year of medical school you completed in the 2014–2015 academic year)

M1 (first year) (1)

M2 (second year) (2)

M3 (third year) (3)

M4 (fourth year) (4)

Other (please describe in the space provided below) (5)

If M1 (first year) Is Selected, Then Skip To Where were you born?If M2 (second year) Is Selected, Then Skip To Where were you born?If M3 (third year) Is Selected, Then Skip To Where were you born?If M4 (fourth year) Is Selected, Then Skip To Where were you born?

Please describe below your year in medical school

Where were you born?

**Table:**

	USA (1)	USA ~ AK (2)	USA ~ AL (3)	USA ~ AR (4)	USA ~ AZ (5)	USA ~ CA (6)	USA ~ CT (7)	USA ~ DC (8)	USA ~ DE (9)	USA ~ FL (10)	USA ~ GA (11)	USA ~ HI (12)	USA ~ IA (13)	USA ~ ID (14)
Country (1)														
State (2)														
	USA ~ IL (15)	USA ~ IN (16)	USA ~ KS (17)	USA ~ KY (18)	USA ~ LA (19)	USA ~ MA (20)	USA ~ MD (21)	USA ~ ME (22)	USA ~ MI (23)	USA ~ MN (24)	USA ~ MO (25)	USA ~ MS (26)	USA ~ MT (27)	USA ~ NC (28)
Country (1)														
State (2)														
	USA ~ ND (29)	USA ~ NE (30)	USA ~ NH (31)	USA ~ NJ (32)	USA ~ NM (33)	USA ~ NV (34)	USA ~ NY (35)	USA ~ OH (36)	USA ~ OK (37)	USA ~ OR (38)	USA ~ PA (39)	USA ~ RI (40)	USA ~ SC (41)	USA ~ SD (42)
Country (1)														
State (2)														
	USA ~ TN (43)	USA ~ TX (44)	USA ~ UT (45)	USA ~ VA (46)	USA ~ VT (47)	USA ~ WA (48)	USA ~ WV (49)	USA ~ WY (50)	-51	~ WY (52)	Afghanistan (53)	Afghanistan ~ N/A (54)	Albania (55)	Albania ~ N/A (56)
Country (1)														
State (2)														

	Algeria (57)	Algeria ~ N/A (58)	Andorra (59)	Andorra ~ N/A (60)	Angola (61)	Angola ~ N/A (62)
Country (1)						
State (2)						

(Table Truncated to 63 Columns)

Where is your hometown?

**Table:**

	USA (1)	USA ~ AK (2)	USA ~ AL (3)	USA ~ AR (4)	USA ~ AZ (5)	USA ~ CA (6)	USA ~ CT (7)	USA ~ DC (8)	USA ~ DE (9)	USA ~ FL (10)	USA ~ GA (11)	USA ~ HI (12)	USA ~ IA (13)	USA ~ ID (14)
Country (1)														
State (2)														

	USA ~ IL (15)	USA ~ IN (16)	USA ~ KS (17)	USA ~ KY (18)	USA ~ LA (19)	USA ~ MA (20)	USA ~ MD (21)	USA ~ ME (22)	USA ~ MI (23)	USA ~ MN (24)	USA ~ MO (25)	USA ~ MS (26)	USA ~ MT (27)	USA ~ NC (28)
Country (1)														
State (2)														

	USA ~ ND (29)	USA ~ NE (30)	USA ~ NH (31)	USA ~ NJ (32)	USA ~ NM (33)	USA ~ NV (34)	USA ~ NY (35)	USA ~ OH (36)	USA ~ OK (37)	USA ~ OR (38)	USA ~ PA (39)	USA ~ RI (40)	USA ~ SC (41)	USA ~ SD (42)
Country (1)														
State (2)														

	USA ~ TN (43)	USA ~ TX (44)	USA ~ UT (45)	USA ~ VA (46)	USA ~ VT (47)	USA ~ WA (48)	USA ~ WV (49)	USA ~ WY (50)	(51)	~ WY (52)	Afghanistan (53)	Afghanistan ~ N/A (54)	Albania (55)	Albania ~ N/A (56)
Country (1)														
State (2)														

	Algeria (57)	Algeria ~ N/A (58)	Andorra (59)	Andorra ~ N/A (60)	Angola (61)	Angola ~ N/A (62)
Country (1)						
State (2						

(Table Truncated to 63 Columns)

How religious do you consider yourself to be? (1 = not at all; 5 = a whole lot)

1 (1)

2 (2)

3 (3)

4 (4)

5 (5)

What sex was assigned to you at birth?

Female (1)

Male (2)

Intersex (3)

With which gender identity do you most identify currently?

Female (1)

Male (2)

Trans woman (3)

Trans man (4)

Genderqueer (5)

Other (6)

Which of the following best describes your sexual orientation identity?

Lesbian (1)

Bisexual (2)

Gay (3)

Queer (4)

Heterosexual (5)

Other (6)

During your life, with whom have you had sexual contact? (Please check all that apply)

Female (1)

Male (2)

Trans woman (3)

Trans man (4)

Genderqueer (5)

Other (6)

I have never had sexual contact (7)

To your knowledge, are any of your family members part of a sexual or gender minority population (e.g. LGBTQ, GNC, born with a DSD)?

Yes (1)

I strongly suspect yes, but I am not sure (2)

No (3)

To your knowledge, are any of your friends part of a sexual or gender minority population (e.g. LGBTQ, GNC, born with a DSD)?

Yes (1)

I strongly suspect yes, but I am not sure (2)

No (3)

Thank you for participating in this survey! If you would like to enter a raffle to win one of ten $25 Amazon gift cards, please follow the link below, or cut and past the link into your browser. None of your contact details submitted for the raffle can be linked to your responses on this survey.https://yalesurvey.qualtrics.com/SE/?SID=SV_er1GmC4kDoN4aDb

## References

[1] Lambda Legal When health care isn’t caring: lambda Legal’s survey of discrimination against LGBT people and people with HIV. New York: Lambda Legal; 2010.

[2] Eliason MJ, Schope R. Does “don’t ask don’t tell” apply to health care? Lesbian, gay, and bisexual people’s disclosure to health care providers. J Gay Lesbian Med Assoc. 2001;5(4):125–14.

[3] [OCEF] One Colorado Education Fund Invisible: the state of LGBT health in Colorado. Denver (CO): One Colorado Education Fund (US); 2011.

[4] James SE, Herman JL, Rankin S, et al 2016 The Report of the 2015 U.S. Transgender Survey. Washington (DC): National Center for Transgender Equality

[5] [IOM] Institute of Medicine Committee on Lesbian, Gay, Bisexual, and Transgender Health Issues and Research Gaps and Opportunities The health of lesbian, gay, bisexual, and transgender people: building a foundation for better understanding. Washington (DC): National Academies of Sciences (US); 2011. 22013611

[6] [AAMC] Association of American Medical Colleges Implementing curricular and institutional climate changes to improve health care for individuals who are LGBT, gender nonconforming, or born with DSD: a resource for medical educators. Washington (DC): Association of American Medical Colleges; 2014.

[7] Obedin-Maliver J, Goldsmith ES, Stewart L, et al Lesbian, gay, bisexual, and transgender-related content in undergraduate medical education. JAMA. 2011;306(9):971–977. 21900137 10.1001/jama.2011.1255

[8] White W, Brenman S, Paradis E, et al Lesbian, gay, bisexual, and transgender patient care: medical students’ preparedness and comfort. Teach Learn Med. 2015;27(3):254–263. 26158327 10.1080/10401334.2015.1044656

[9] U.S. Census Bureau [Internet] Washington (DC): U.S. Census Bureau; c1998–2013 [updated 2013; cited 2013; cited 2016 1210]. Available from: https://www2.census.gov/geo/pdfs/maps-data/maps/reference/us_regdiv.pdf

[10] [AAMC] Association of American Medical Colleges Medical student graduation questionnaire: 2015. Vol. 2015; Washington, DC: Association of American Medical Colleges; 2015.

[11] Lapinski J, Sexton P, Baker L Acceptance of lesbian, gay, bisexual, and transgender patients, attitudes about their treatment, and related medical knowledge among osteopathic medical students. J Am Osteopath Assoc. 2014;114(10):788–796. 25288714 10.7556/jaoa.2014.153

[12] Pew Research Center The American-Western European values gap. Washington (DC): Pew Charitable Trusts (US); 2011.

[13] Pew Research Center The global divide on homosexuality. Washington (DC): Pew Charitable Trusts (US); 2013.

[14] Sánchez NF, Rankin S, Callahan E, et al LGBT trainee and health professional perspectives on academic careers–facilitators and challenges. LGBT Health. 2015;2(4):346–356. 26788776 10.1089/lgbt.2015.0024

[15] Hardeman RR, Przedworski JM, Burke SE, et al Mental well-being in first year medical students: a comparison by race and gender: a report from the medical student CHANGE study. J Racial Ethn Health Disparities. 2015;2(3):403–413. 26413458 10.1007/s40615-015-0087-x PMC4579540

[16] Blanch-Hartigan D Medical students’ self-assessment of performance: results from three meta-analyses. Patient Educ Couns. 2011;84(1):3–9. 20708898 10.1016/j.pec.2010.06.037

[17] Cheng LF, Yang HC Learning about gender on campus: an analysis of the hidden curriculum for medical students. Med Educ. 2015;49(3):321–331. 25693991 10.1111/medu.12628

[18] Oancia T, Bohm C, Carry T, et al The influence of gender and specialty on reporting of abusive and discriminatory behavior by medical students, residents and physician teachers. Med Educ. 2000;34(4):250–256. 10733720 10.1046/j.1365-2923.2000.00561.x

[19] Holm AL, Rowe GM, Brady M, et al Recognizing privilege and bias: an interactive exercise to expand health care providers’ personal awareness. Acad Med. 2016;3:360–364. 10.1097/ACM.0000000000001290 27355785

[20] Schatz B, O’Hanlan KA Anti-gay discrimination in medicine: results of a national survey of lesbian, gay and bisexual physicians. San Francisco (CA): Gay and Lesbian Medical Association (US); 1994.

[21] Cooper-Patrick L, Gallo JJ, Vu HT, et al Race, gender, and partnership in the patient-physician relationship. JAMA. 1999;282(6):583–589. 10450723 10.1001/jama.282.6.583

[22] Hall W, Violato R, Lewkonia J, et al Assesment of physician performance in Alberta: the physician achievement review. Can Med Assoc J. 1999;161(1):52–57. 10420867 PMC1232653

[23] Saha S, Komaromy M, Koepsell T, et al Patient-physician racial concordance and the perceived quality and use of health care. Arch Intern Med. 1999;159(9):997–1004. 10326942 10.1001/archinte.159.9.997

[24] Kelley L, Chou CL, Dibble SL, et al A critical intervention in lesbian, gay, bisexual, and transgender health: knowledge and attitude outcomes among second-year medical students. Teach Learn Med. 2008;20(3):248–253. 18615300 10.1080/10401330802199567

[25] [OCEF] One Colorado Education Fund Becoming visible: working with Colorado physicians to improve LGBT health. Denver (CO): One Colorado Education Fund (US); 2013.

[26] Burke SE, Dovidio JF, Przedworski JM, et al Do contact and empathy mitigate bias against gay and lesbian people among heterosexual medical students? A report from Medical Student CHANGES. Acad Med. 2015;90(5):645–651. 25674910 10.1097/ACM.0000000000000661 PMC4414697

[27] Gates GJ LGBT demographics: comparisons among population-based surveys. Los Angeles (CA): Williams Institute, UCLA School of Law (US); 2014.

[28] Sanchez NF, Rabatin J, Sanchez JP, et al Medical students’ ability to care for lesbian, gay, bisexual, and transgendered patients. J Fam Med. 2006;38(1):21–27. 16378255

[29] Hardacker CT, Rubinstein B, Hotton A, et al Adding silver to the rainbow: the development of the Nurses’ Health Education About LGBT Elders (HEALE) cultural competency curriculum. J Nurs Manag. 2014;22(2):257–266. 23869475 10.1111/jonm.12125

[30] [AAMC] Association of American Medical Colleges: sexual and gender minority health resources [Internet]. Washington (DC): Association of American Medical Colleges; c2016 [cited 2016 1210]. Available from: https://www.aamc.org/initiatives/diversity/lgbthealthresources/

[31] Snowdon S The medical school curriculum and LGBT health concerns. Virtual Mentor. 2010;12(8):638–643. 23186847 10.1001/virtualmentor.2010.12.8.medu1-1008

[32] Blaney S, Gardner S, Garuz J, et al 2013 Analysis of learning outcomes in LGBTQ medical school curriculum. Paper presented at: 141st APHA Annual Meeting and Exposition; Boston, MA.

[33] [AAMC] Association of American Medical Colleges Leading change applications of the AAMC LGBT, gender nonconforming, and DSD health resources in academic health centers. AAMC Videos and Resources. Washington (DC): Association of American Medical Colleges (US); 2015 Available from: https://www.aamc.org/initiatives/diversity/460168/leadingchangeapplications.html

[34] Wilson CK, West L, Stepleman L, et al Attitudes toward LGBT patients among students in the health professions: influence of demographics and discipline. LGBT Health. 2014;1(3):204–211. 26789713 10.1089/lgbt.2013.0016

[35] White W, Brenman S, Paradis E, et al Lesbian, gay, bisexual, and transgender patient care: medical students’ preparedness and comfort. Teach Learn Med. 2015;27(3):254–263. 26158327 10.1080/10401334.2015.1044656

[36] Lapinski J, Sexton P, Baker L Acceptance of lesbian, gay, bisexual, and transgender patients, attitudes about their treatment, and related medical knowledge among osteopathic medical students. J Am Osteopath Assoc. 2014 10;114(10):788–796. 25288714 10.7556/jaoa.2014.153

[37] Sanchez NF, Rabatin J, Sanchez JP, Hubbard S, Kalet A Medical students’ ability to care for lesbian, gay, bisexual, and transgendered patients. Fam Med. 2006 1;38(1):21–27. 16378255

[38] Nama N, MacPherson P, Sampson M, HJ McMillan Medical students’ perception of lesbian, gay, bisexual, and transgender (LGBT) discrimination in their learning environment and their self-reported comfort level for caring for LGBT patients: a survey study. Med Educ Online. 2017;22(1):1368850. 28853327 10.1080/10872981.2017.1368850 PMC5653936

[39] JJ Liang, IH Gardner, JA Walker, JD Safer Observed deficiencies in medical student knowledge of transgender and intersex health. Endocr Pract. 2017 8;23(8):897–906. Epub 2017 May 23. 28534684 10.4158/EP171758.OR

[40] Beck Dallaghan G.L., Medder J., Zabinski J. et al. Lesbian, Gay, Bisexual, and Transgender Health: a Survey of Attitudes, Knowledge, Preparedness, Campus Climate, and Student Recommendations for Change in Four Midwestern Medical Schools. Med Sci Educ. 2018 DOI:10.1007/s40670-018-0536-3

